# Radiofrequency‐assisted subcision combined with polycaprolactone‐based dermal fillers in the management of atrophic facial acne scars: A pilot investigative study

**DOI:** 10.1111/srt.13228

**Published:** 2022-11-01

**Authors:** Elaheh Lotfi, Mohammad Shafie'ei, Najmeh Ahramiyanpour

**Affiliations:** ^1^ Yousef Abad Skin and Hair Center Tehran Iran; ^2^ Faculty of Medicine Kerman University of Medical Sciences Kerman Iran; ^3^ Department of Dermatology Afzalipour Hospital, Afzalipour Faculty of Medicine, Kerman University of Medical Sciences Kerman Iran; ^4^ Pathology and Stem Cell Research Center Kerman University of Medical Sciences Kerman Iran

**Keywords:** atrophic acne scars, collagen stimulation, polycaprolactone‐based fillers, radiofrequency‐assisted subcision

## Abstract

**Objective:**

The scar's appearance and psychological burden are the most esthetically challenging issues in acne vulgaris. This study investigated the effectiveness and safety profile of combined radiofrequency‐assisted subcision, which, even though effective on both Icepeak and Rolling subtypes, is only mildly effective on boxcar lesions, and polycaprolactone‐based dermal filler with collagen stimulation potency in managing atrophic postacne scars.

**Methods:**

Our quasi‐experimental single‐arm study, after the inclusion of 10 cases over the age of 18 with moderate to severe mixed atrophic facial acne scarring, was carried out in two 3‐month separate sessions, during and after the intended intervention of which the experienced lesion improvements and also adverse events were documented. Moreover, all cases were followed for 3 months after the last session, evaluating the differences in the mentioned outcomes.

**Results:**

We found the combined intervention to be highly effective in improving the intended outcomes, with the total number of acne lesions (*p*‐value < 0.001), along with the total number of Ice peak (*p*‐value = 0.002), Rolling (*p*‐value < 0.001), and boxcar (*p*‐value = 0.023) lesions demonstrating statistically significant changes.

**Conclusion:**

Radiofrequency‐assisted subcision combined with polycaprolactone‐based dermal fillers can be an effective intervention in managing postacne scars. However, we recommend that randomized clinical trials with larger sample sizes be carried out for a more precise conclusion.

## 1 INTRODUCTION

The scar appearance resulting from deep‐lying inflammatory acne and its subsequent psychological burden is the most esthetically challenging issue in acne vulgaris disease.^1^ These scars have many well‐understood morphologies but are generally divided into two main subtypes of hypertrophic and atrophic, with the latter (consisting of rolling, icepeak, and boxcar subtypes) being the more common of the two.^2^ Managing these scars has also proven to rely on many factors, including the patient's adherence and expectations while accounting for the clinician's initial evaluations.^3^ Therefore, these factors, in aggregate, have caused the desired esthetic outcome of these scars to be very challenging.

Although most beneficial for the atrophic subtype of scars, one management technique is the subcutaneous incision, termed subcision, which has led to a renewal of the wound healing process and collagen synthesis.^4^, ^5^, ^6^ However, even though the advantages, including shortened required patient downtime and fewer adverse effects on the adjacent melanin, are very eye‐catching, the side effects, including mechanically induced tissue trauma and the resulting hemorrhage, which in some cases can further complicate the situation by forming large hematomas and fibrosis, are left as the main worries.^7^ These adverse events have notably decreased when techniques, including radiofrequency (i.e., radiofrequency‐assisted subcision, RF‐subcision, or rsubcision; using thermal energy to aid in the cannula movement), complementary to conventional subcision are implemented.^7^, ^8^, ^9^ Moreover, studies have also demonstrated additional positive effects when the technique is used.^7^, ^9^, ^10^, ^11^

With all the merits, however, reports have indicated that boxcar acne scars were the toughest to resolve following the utilization of the technique.^9^ Therefore, we believe the improvement in the outcome could be further increased by utilizing potentially effective complementary interventions, including dermal fillers with collagen‐stimulating potency.^12^, ^13^

One of the available mentioned fillers is the polycaprolactone‐based dermal fillers, demonstrating their efficacy in stimulating the production of collagens with higher endurance and esthetic properties while also improving the depressed appearance, which had remained postinjury.^14^, ^15^, ^16^, ^17^, ^18^, ^19^, ^20^, ^21^ Moreover, even though the effectiveness of these fillers has not yet been studied on atrophic acne scarring, their rejuvenating capabilities have been demonstrated to be significant when their application was investigated on the striae distensae disappearance, nasolabial folds' correction, and forehead contouring.^22^, ^23^, ^24^, ^25^ All of the mentioned favorable results further encouraged us to put our hypothesis to the test. Therefore, this study aimed to evaluate the differences the said addition would lead to in managing atrophic acne scars.

## 2 METHODS

### 2.1 Study design and ethical considerations

After being ethically approved by the local ethics committee, our pilot single‐arm study was conducted from July 2021 to February 2022 in a clinic with an outpatient setting while following the ethical principles of the Declaration of Helsinki. Furthermore, written informed consent was obtained from all participants before the study began.

### 2.2 Participant eligibility criteria

We opted to define our criteria based on the population, intervention, comparator, and outcome framework to achieve the highest possible degree of uniformity and objectiveness in selecting participants and the conduction methodology.^26^ However, the framework's comparator aspect was not considered due to our study's design.

### 2.3 Population

Due to the novelty of the intervention and a lack of a prior study on the outcomes' mean differences, the determination of a statistically sufficient sample size was not possible. Therefore, a sample consisting of 10 individuals was deemed convenient.

Individuals over 18 who were referred with a complaint of facial acne scars made up our study population. Our inclusion criteria were those suffering from moderate to severe mixed (i.e., ice peak, boxcar, and rolling subtypes) atrophic postacne scarring. Furthermore, individuals who had underlying diseases that could affect the outcomes of the study were excluded, including:

1. The presence of cardiac pacemakers or metal implants in the body,
2. Those with active acne lesions or superimposed infections, or
3. Participants who had received other possibly effective therapeutic methods (e.g., laser therapy or chemical or physical peeling techniques) in the study's 4 months of the treatment phase.

### 2.4 Intervention

Regarding the RF‐subcision, the methodology was as follows: first, after appropriate sterilizations, the scar studded areas were anesthetized via a local lidocaine 2% gel application without requiring a tumescent injection or neural blocking. Next, the radiofrequency device (Aphrodite, Iran; electrosurgical unit, 400 mA current, maximum output power of 60 watts, and operating frequency of 4 MHz) was utilized, with the neutral plate being connected to the chest after applying a lubricant gel. Next, the power was set to 30–40 watts to achieve the most suitable temperatures (30‐40 degrees centigrade at dermis) and outcomes, as higher powers would lead to the burning of the skin. Depending on the severity of scarring, either the 18 (the most commonly used), 19, or 21 gauge needle, with the bevel pointing upwards and connected through the opposing end to the monopolar cannula, superficially infiltrated the lesion at a 30–45 degree angle from a periauricular entry point 1 centimeter away from the scar, the depth of insertion of which ranged from mid to deep dermis depending on the lesion's severity. Afterward, the entangled mid to deep dermal fibrous bands were released with a rapid parallel‐to‐skin to‐and‐fro motion while holding down the device's pedal. Furthermore, the area was intermittently monitored to prevent excessive heating and thermal damage to the neighboring tissues. After the intended procedure, the pedal was released, the RF probe removed from the opposite end, the cannula was retrieved, and a sterile gauze was placed adjacent to the entry site to prevent burning.

In each session, a total of 0.5 ml (0.05–0.1 ml of filler in each lesion) of the polycaprolactone‐based dermal filler (Ellansé, Netherlands) was also superficially injected with a 27 gauge needle, via fanning mode, into the deep dermis of the scars. Then, the skin surface was massaged repeatedly to achieve and maintain the desired esthetic and appearance. Additionally, the priorly mentioned photographic assessments and scores were obtained before the session.

In addition, we tried to mitigate the unnecessary infectious and painful side effects by administering topical antibiotics and nonsteroidal anti‐inflammatory drugs for a maximum of 3 days. Moreover, the cases received sunscreen agents to prevent damage to the vulnerable area between the sessions.

### 2.5 Outcomes

At first, in addition to the demographic data (i.e., the age and sex of each case), the scar characteristics, including numbers, type (ice peak, rolling, or boxcar), and the severity grading, which was based on the Goodman and Baron's qualitative^27^ (scored from mild to severe based on whether they are apparent in a distance of 50 cm and above, being covered using make‐up, and being flattened by manual stretching of the skin, respectively) and quantitative^28^ (achieved by two independently assessing dermatologists on a scale based on the scar count and type from 0 to a theoretical upper limit of 84; on which mild scarring can score to a maximum of 6 points, moderate scarring potentially scoring up to a maximum of 18 points, and severe scarring being capable of going as high as 36 on the overall scores) Global Acne Scarring Grading Systems (GBGS) were documented as measures of the primary outcome at baseline and 3 months after the last therapy session. Furthermore, the considered lesions were photographed (Sony A7Rii, 42 megapixels, Tokyo, Japan) and appropriately anonymized. Moreover, the secondary outcome of interest, consisting of any noted immediate postprocedural adverse reactions or adverse events during the follow‐up sessions, was documented.

### 2.6 Follow‐up

Individuals who underwent the therapy session were then followed for 6 months (i.e., three months after the second therapy session), during which the resulting changes in the appearance, grading, and the number of lesions were photographed and also anonymized.

### 2.7 Statistical analysis

The 26th version of the SPSS statistical package (SPSS Inc., Chicago, IL, USA) was used for the analyses. *p*‐Values <0.05 were considered statistically significant.

Initially, the normality of the data distribution was determined by the One‐Sample Kolmogorov‐Smirnov test. The consistency in the assessors’ ratings before and after the intervention was also evaluated by determining the Spearman's rank correlation coefficient (*ρ*).

Paired samples *t*‐test was performed to compare the means of the acquired data with a normal distribution (i.e., the total number of acne lesions, total number of Ice peak lesions, and total number of rolling lesions), while the Wilcoxon rank test was performed for the analysis of the data with without normal distribution (i.e., the total number of boxcar lesions and the changes in the mean of the grading of the Goodman and Baron's score by the two dermatologists).

## 3 RESULTS

A total of 10 individuals were included in our investigation, seven of whom (70%) were female, averaging 31.3 ± 0.83 years. Moreover, all the included cases completed the study. These individuals had a mean total number of facial acne lesions of 18.70 ± 9.056 (range: 9–35), consisting, in order of frequency, of ice peak (9.7 ± 7.65; range: 2–22), rolling (7 ± 2.70; range: 2–11), and boxcar (2 ± 1.700; range: 0–5 with 20% of the participants not having any boxcar lesions while 50% had two or more lesions) lesions, respectively. Eight individuals suffered from severe postacne scars, while the remaining two had moderate grades. (Tables 1 and 2)

**TABLE 1:** Demographic data of the included individuals at baseline

**Age**	
Mean +/‐ SD	31.3 ± 0.83
Range	28–37
**Gender**	
Male (%)	3 (30%)
Female (%)	7 (70%)
**Fitzpatrick skin type**	
III	6
IV	4

**TABLE 2:** Changes in the number of acne lesions before and following the procedure

Variable	Baseline	After the procedure	Mean difference (SD)	*p*‐Value
Total number of acne lesions (mean ± SD)	18.70 ± 9.056	6.80 ± 4.34	11.90 (5.53)	<0.001
Mean total number of ice peak lesions (mean ± SD)	9.7 ± 7.65	4 ± 3.74	5.70 (4.24)	0.002
Mean total number of rolling lesions (mean ± SD)	7 ± 2.70	1.80 ± 0.79	5.200 (2.251)	<0.001
Total number of boxcar lesions (mean ± SD)	2 ± 1.700	1 ± 0.943	1 (0.943)	0.023

**FIGURE 1:**
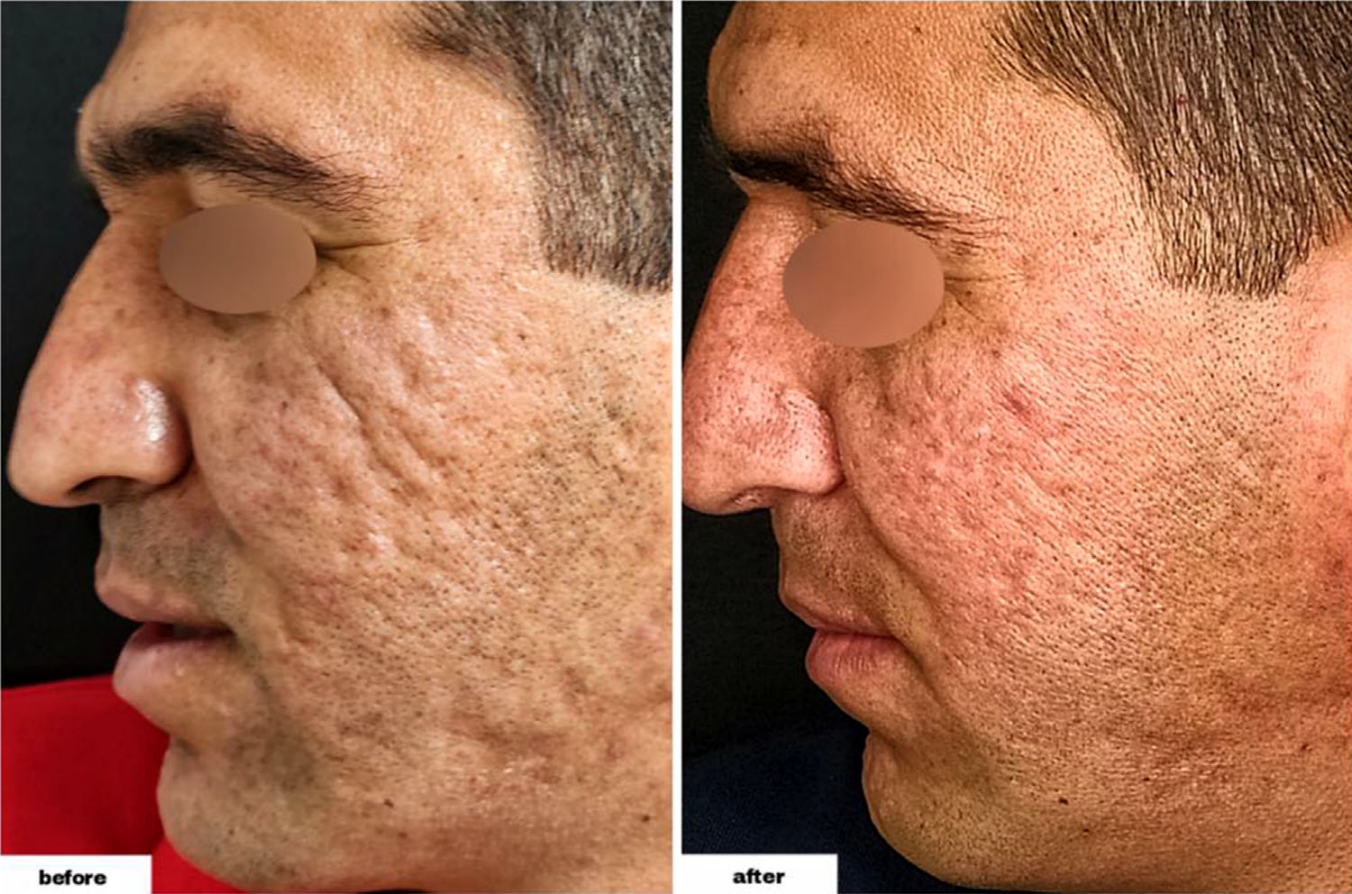
The scar appearance in a 34‐year‐old male before and after the procedure

**FIGURE 2:**
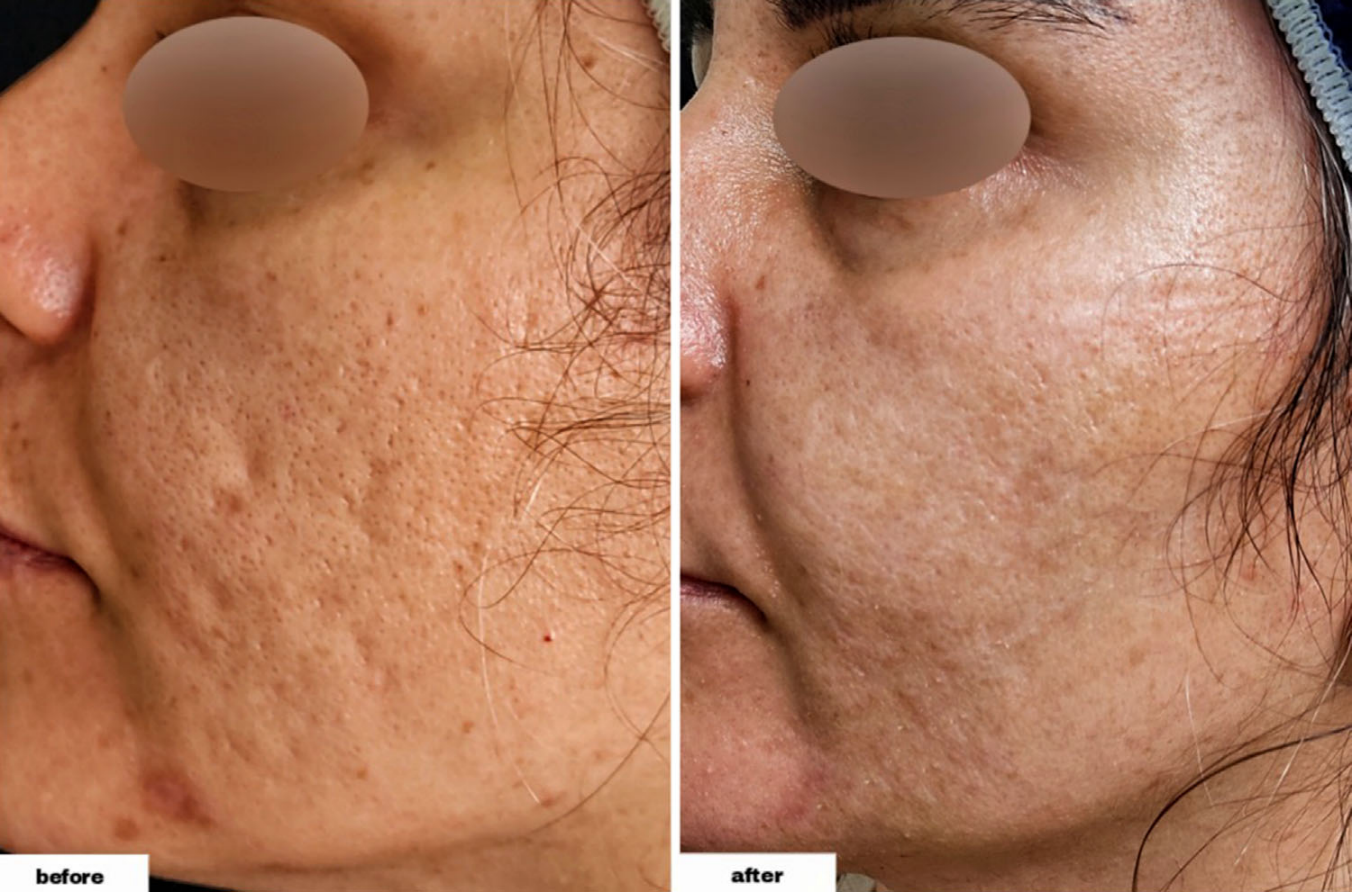
The procedure's effectiveness in decreasing the number of Rolling scars in a 37‐year‐old female

**FIGURE 3:**
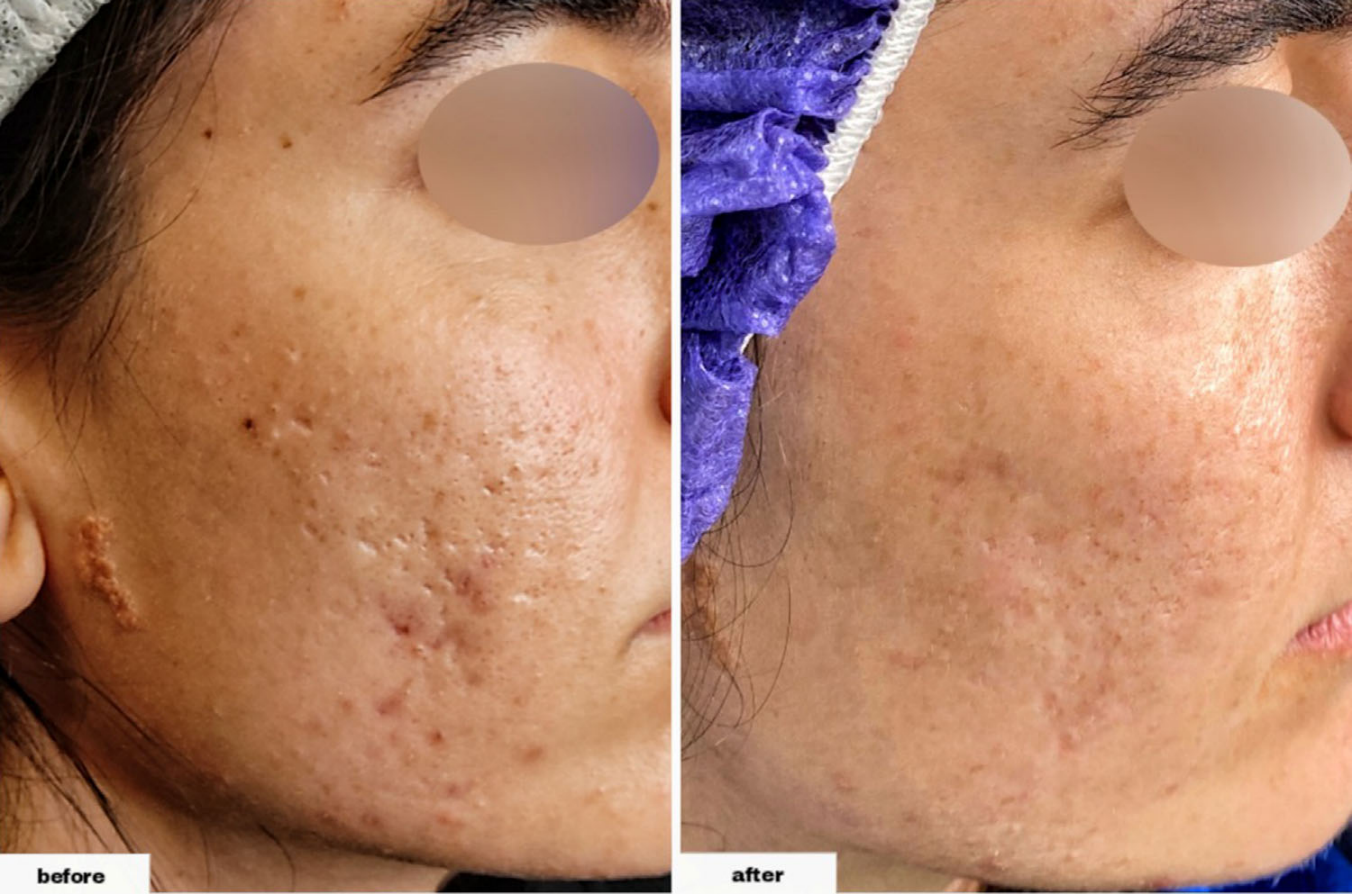
The procedure's effectiveness in decreasing the number of Ice peak scars in a 28‐year‐old female

**FIGURE 4:**
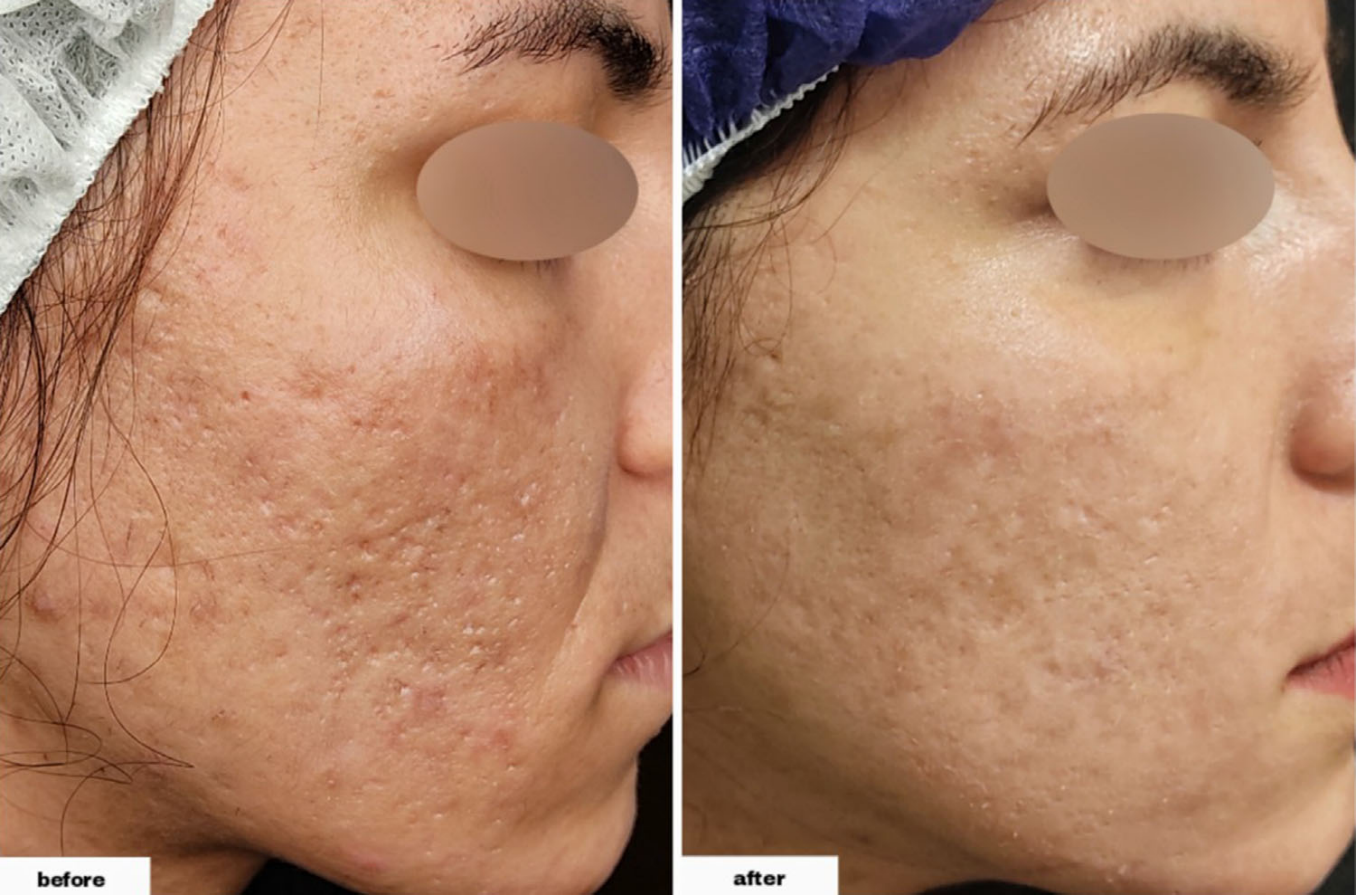
The procedure's effectiveness in decreasing the number of boxcar scars in a 29‐year‐old female

As demonstrated in Table 2, we found that the differences were significant in all types of lesions following the procedure. The total number of acne lesions (postintervention range: 3–15) decreased by 11.90 ± 5.53 (*p*‐values of <0.001). Furthermore, the total number of ice peak lesions (postintervention range: 0–12) decreased by 5.70 ± 4.24 (*p*‐value = 0.002), the total number of rolling lesions (postintervention range: 1–3) decreased by 5.20 ± 2.25 (*p*‐value < 0.001), and the total number of boxcar lesions (postintervention range: 0–3, with 30% of individuals having zero number of the lesion and 80% overall having one or less) decreased by 1 ± 0.94 (*p*‐value = 0.023). (Figures 1, 2, 3, 4)

The *ρ* values indicating the level of association between the two ratings before and after the intervention were 0.985 and 0.972, respectively, which shows a strong agreement. Moreover, the qualitative and quantitative grading of scars at the end of the investigations indicated significantly favorable responses (*p*‐values of 0.004 and <0.001, respectively), as all individuals experienced an improvement of at least one grade in the severity of scarring. This finding meant that eight individuals' scarring severity improved to mild (six and two cases with baseline grades indicating severe and moderate scarring, respectively), while two improved to moderate (having severe scarring at baseline). Moreover, this finding translated to a mean improvement of 11.4 ± 3.61 in the quantitative scoring system (Table 3).

**TABLE 3:** Goodman and Baron's qualitative and quantitative scores before and after the intervention

Variable		Baseline	After the procedure	*p*‐Value
Goodman & Baron's Qualitative Score	**Mild**	0	8	0.004*
	**Moderate**	2	2	
	**Severe**	8	0	
Goodman & Baron's quantitative score (mean ± SD)	**Baseline**	**After the procedure**	**Mean difference**	<0.001*
	19.3 ± 5.31	7.9 ± 4.06	11.4 ± 3.61	

* Analyzed via the Wilcoxon rank test

Regarding the safety profile of the intervention, we found that the individuals primarily complained of mild localized edema (30% of individuals and lasting for a maximum of 1 week), mild ecchymosis (30% of individuals and lasting for a maximum of 1 week), mild burning sensation (10% of individuals), and hypoesthesia (10% of individuals and lasting for a maximum of 3 days) during therapy. However, these complaints did not lead to said individuals abstaining from the study.

## 4 DISCUSSION

Many studies have investigated whether adding any supplementary techniques or agents would address concerns arising from postacne scars, including their quality of life and mental health.^29^ In our study, which, based on our online database search, has been carried out for the first time, we demonstrated that in a study group of majority women individuals suffering from postacne scarring, the relatively novel approach, consisting of a combination of Radiofrequency‐subcision and the polycaprolactone‐based dermal filler, significantly affected the scars’ numbers and appearance through the radiofrequency‐induced heat.

We found that the total number of acne scars not only decreased significantly, and the qualitative and quantitative GBGS gradings indicated highly significant improvements, but so did the three subtypes (rolling, icepeak, and boxcar) individually. This finding indicates that the combined method can potentially improve a broad range of acne scars and not be bound to what had previously hampered the ultimate esthetic outcome of the disease in many individuals. These findings are consistent with the study by Kaur et al.^9^ , investigating the effectiveness of RF‐subcision versus conventional subcision. However, our study showed the combined interventions’ superior practical effectiveness in improving the number of boxcar scars compared to RF‐subcision alone, which was improved, although significantly due to the low number of the lesion, but to a lesser extent and only by a few.

The technique also only caused mild to moderate adverse events, including mild localized edema, ecchymosis, and slight hypoesthesia during therapy, most of which were temporary and resolved either during the study or at the end. Furthermore, even though the lattermost complaint was relatively disconcerting for the individuals who underwent the therapy regimen, the positive outcomes outweighed them encouragingly, prompting them to go through the intervention phase and not drop out.

However, our study suffered from a few limitations, including a low number of participants. Therefore, as the results of our research can be invigorating to many health‐seeking individuals and the relevant clinicians, we recommend that further studies be carried out with the primary intention of addressing the mentioned limitations our study faced. As more similar data become available, we believe that the definite conclusion would incentivize the scientific community greatly.

## 5 CONCLUSION

The combined intervention of RF‐subcision and polycaprolactone‐based fillers can be regarded as one of the effective and safe methods in managing the acne‐induced atrophic scar appearance and its various lesion subtypes, including boxcar. Moreover, the high adherence rate demonstrated in the study can also be very encouraging.

## CONFLICT OF INTEREST

The authors declare that no conflict of or competing interests existed or occurred in the conduction of this manuscript.

## FUNDING INFORMATION

No funding or sponsorship was received for the conduct of this study.

## ETHICS STATEMENT

This study was approved by the Ethics Committee of Kerman University of Medical Sciences under the license of IR.KMU.AH.REC.1400.245. Written informed consent was also obtained from all the participants before the study began.

## Data Availability

The dataset supporting the conclusions of this article is available upon request to the corresponding author, Najmeh Ahramiyanpour.
